# Supplementary material for: Clonal dynamics of germinal center refueling by secondary immunization
Source: bioRxiv. 2026 May 29:2026.05.26.728008. Preprint. [Version 1] doi: 10.64898/2026.05.26.728008 (PMC13232076; doi:10.64898/2026.05.26.728008)
Supplement: 5 [file NIHPP2026.05.26.728008v1-supplement-5.pdf]

bioRxiv preprint doi: https://doi.org/10.64898/2026.05.26.728008; this version posted May 29, 2026. The copyright holder for this preprint (which was not certified by peer review) is the author/funder, who has granted bioRxiv a license to display the preprint in perpetuity. It is made available under a CC-BY 4.0 International license.

# SUPPLEMENTAL FIGURES AND LEGENDS

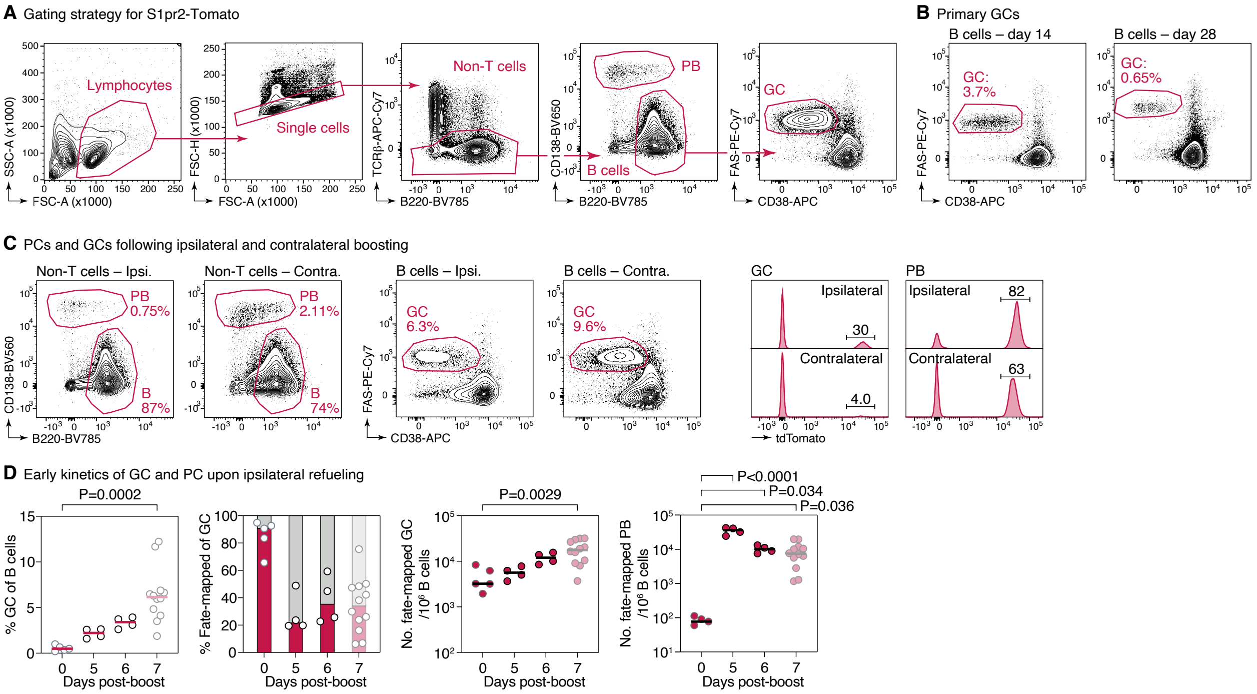


**Figure S1. Response to refueling in mouse LNs after SARS-CoV-2 spike mRNA-LNP immunization. (A)** General gating strategy S1pr2-Tomato fate-mapping. **(B)** Representative flow cytometry plots showing persistence of mRNA-LNP-induced GCs at the time of refueling. Gated on B cells as in (A). **(C)** Representative flow cytometry plots showing the effects of ipsilateral and contralateral on plasmablast (right) and GC B cell (center) numbers and on the fraction of fate-mapped cells within these two populations. Quantified in Fig. 1B,C. **(D)** Evolution of GC and PC expansion upon boosting. Based on flow cytometry data as in (A-C). Each symbol represents one mouse. Data for the day 7 time point (lighter symbols) are reproduced from Fig. 1B,C for comparison.

bioRxiv preprint doi: https://doi.org/10.64898/2026.05.26.728008; this version posted May 29, 2026. The copyright holder for this preprint (which was not certified by peer review) is the author/funder, who has granted bioRxiv a license to display the preprint in perpetuity. It is made available under a CC-BY 4.0 International license.

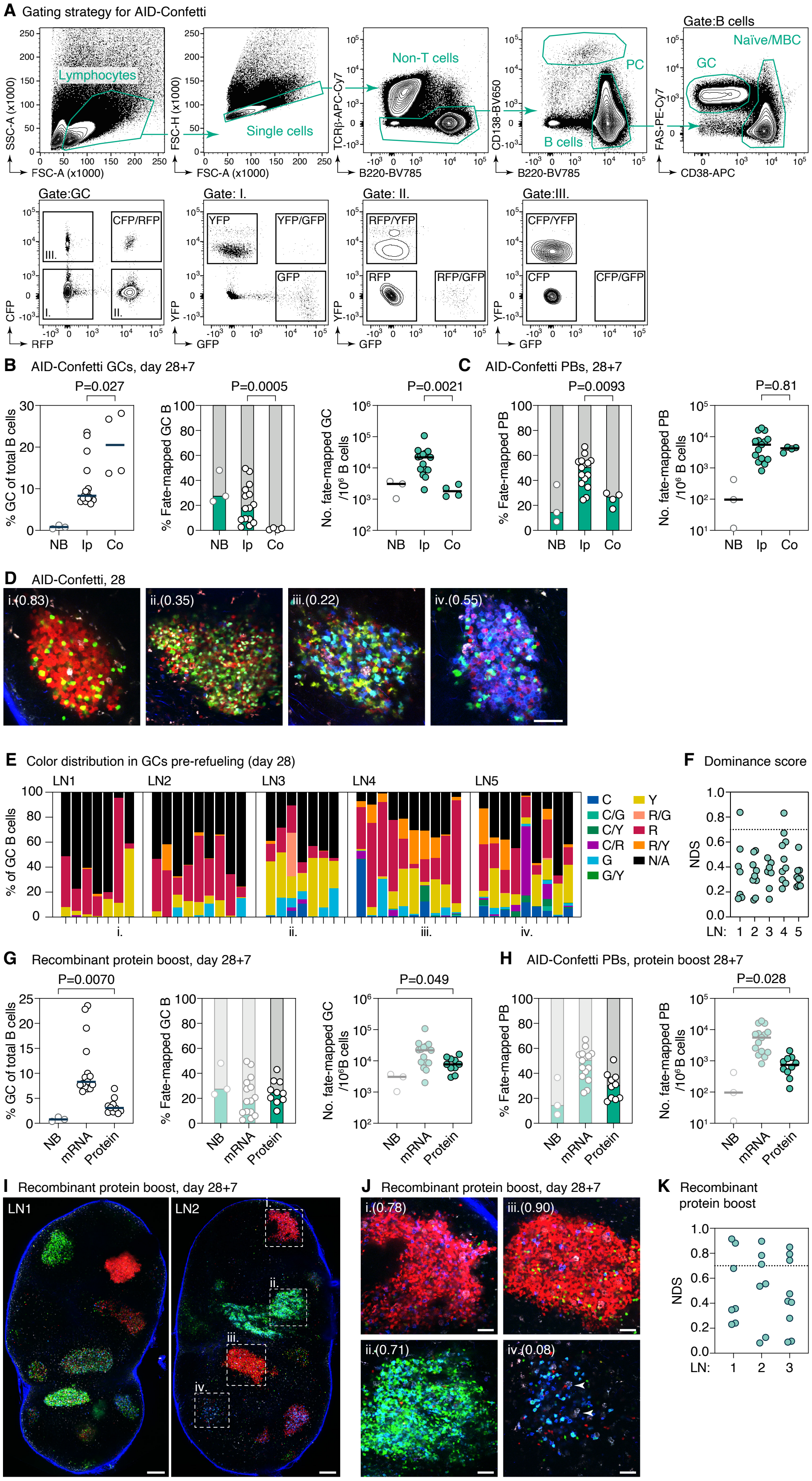

bioRxiv preprint doi: https://doi.org/10.64898/2026.05.26.728008; this version posted May 29, 2026. The copyright holder for this preprint (which was not certified by peer review) is the author/funder, who has granted bioRxiv a license to display the preprint in perpetuity. It is made available under a CC-BY 4.0 International license.

**Figure S2. Tracking GC refueling using Aid-Confetti mice. (A)** General gating strategy for AID-Confetti fate-mapping. **(B)** Quantification of flow cytometry of LN samples generated as in Fig. 1A, using AID-Confetti mice. Graphs show GC size (left), fraction (center), and number (right) of fate-mapped GC B cells. **(C)** As in (B), showing fraction (left) and number (right) of fate-mapped PB. NB, non-boosted; Ip, ipsilateral refueling; Co, contralateral boosting. Each symbol represents one mouse. P-values are for Mann-Whitney U test, only relevant comparisons are shown. **(D)** Representative multiphoton images of and single GCs from AID-Confetti mice at day 28 post-prime, prior to refueling. Details as in Fig. 2B. Scalebars, 50 µm. Numbers in parentheses are NDS. **(E)** Color distribution in individual GCs generated as in (D). Each bar represents one GC, colored by the fraction of cells expressing each color combination (color labels as in Fig. 2C). Roman numerals indicate GCs depicted in (D). **(F)** NDS for GCs in (D,E). Each symbol represents one GC. The dotted line represents a score of 0.7, above which a GC is regarded as a containing a clonal burst. **(G,H)** Quantification of flow cytometry as in (B,C), comparing mice primed with SARS-CoV-2 spike mRNA-LNP and refueled with either mRNA-LNP or recombinant spike protein. Data for NB and mRNA (lighter symbols) are reproduced from panels (B,C) for comparison. **(I,J)** Representative multiphoton images of cross-sections of whole LNs (I) and single GCs (J) from AID-Confetti mice at 7 days post-boosting with recombinant spike protein. Details as in Fig. 2A,B. Scalebars, 200 µm (I) and 50 µm (J). Numbers in parentheses are NDS. **(K)** NDS for GCs in (I,J). Each symbol represents one GC. The dotted line represents a score of 0.7, above which a GC is regarded as a containing a clonal burst.

———

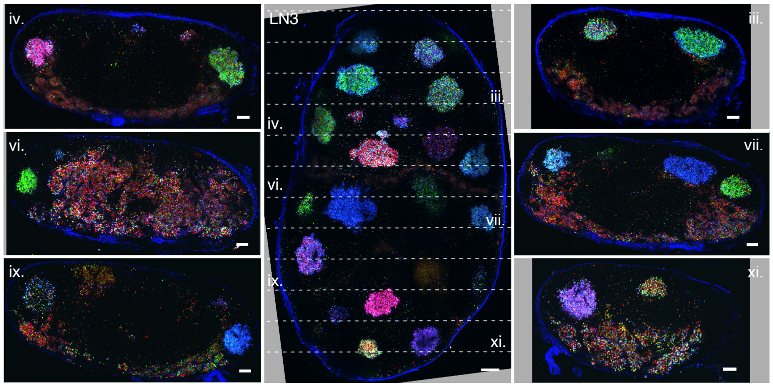


**Figure S3. Multiphoton images exemplifying the process of LN sectioning for single-GC isolation.** The central panel shows an intact GC (reproduced from Fig. 2A) prior to sectioning; panels on the right and left are LN slices imaged post-sectioning. Slices are numbered with roman numerals from the top. Scalebars, 200 µm (overview) and 100 µm (sections).

bioRxiv preprint doi: https://doi.org/10.64898/2026.05.26.728008; this version posted May 29, 2026. The copyright holder for this preprint (which was not certified by peer review) is the author/funder, who has granted bioRxiv a license to display the preprint in perpetuity. It is made available under a CC-BY 4.0 International license.

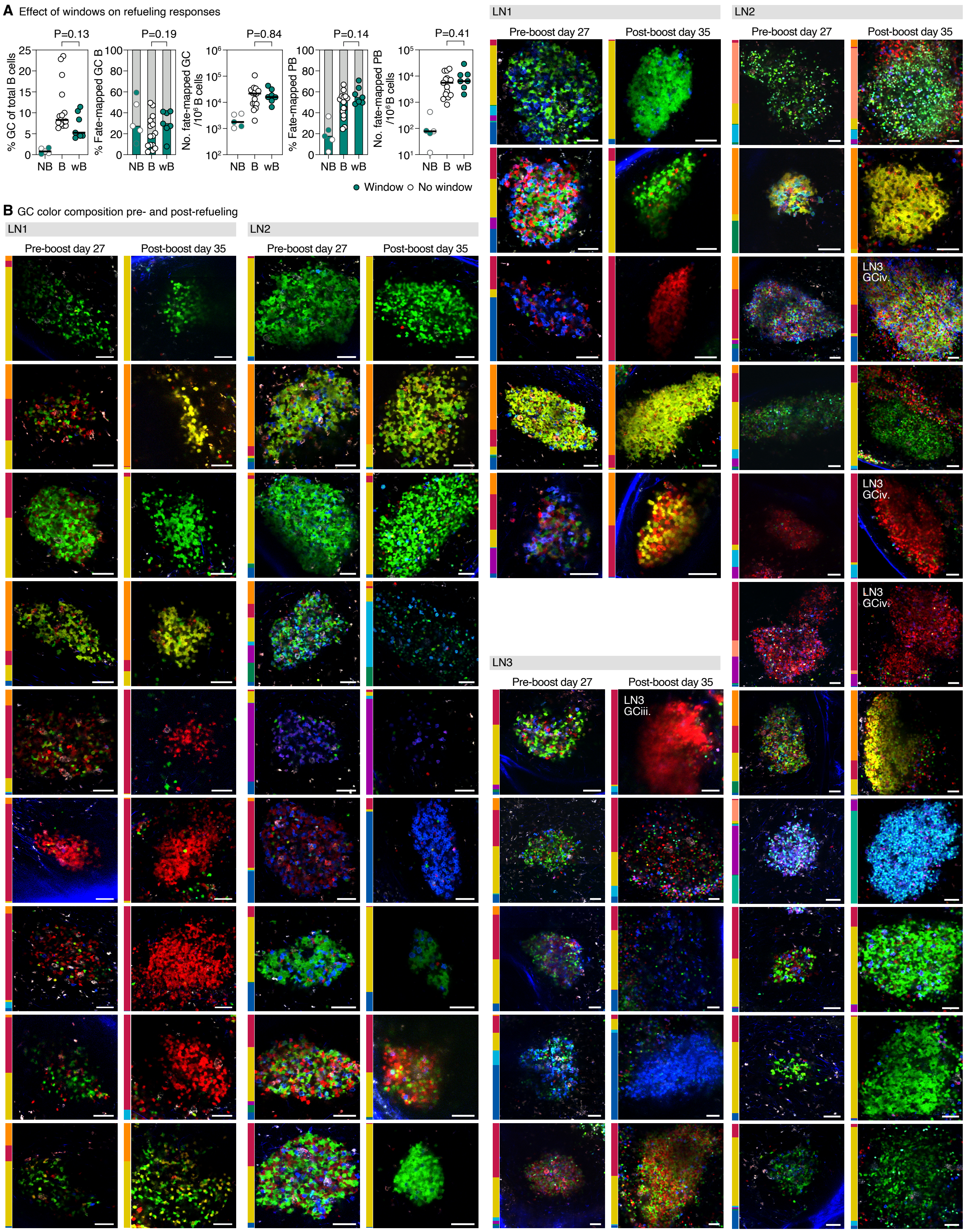

bioRxiv preprint doi: https://doi.org/10.64898/2026.05.26.728008; this version posted May 29, 2026. The copyright holder for this preprint (which was not certified by peer review) is the author/funder, who has granted bioRxiv a license to display the preprint in perpetuity. It is made available under a CC-BY 4.0 International license.

**Figure S4. Imaging GCs sequentially with implanted inguinal LN windows. (A)** Window implantation does not affect the response to refueling. Quantification of flow cytometry as in Fig. S2A,B, comparing the refueling response in mice with and without implanted windows. NB, non-boosted; B, boosted; wB, boosted following implantation and removal of imaging window as described in Fig. 3D. Each symbol represents one mouse. Dark symbols in NB are non-boosted GCs imaged 8 days following window implantation and removal. Data for non-window mice is reproduced from Fig. S2B for comparison and is shown in white symbols. **(B)** Full set of pre-/post-refueling pairs as in Fig. 3E (including those already shown in that image). Bars to the right of each image indicate the fraction of B cells of each fluorescent color in the adjacent image (color labels as in Fig. 2C). LN/GC names are indicated for GCs used for phylogenetic analysis in Fig. 4C.

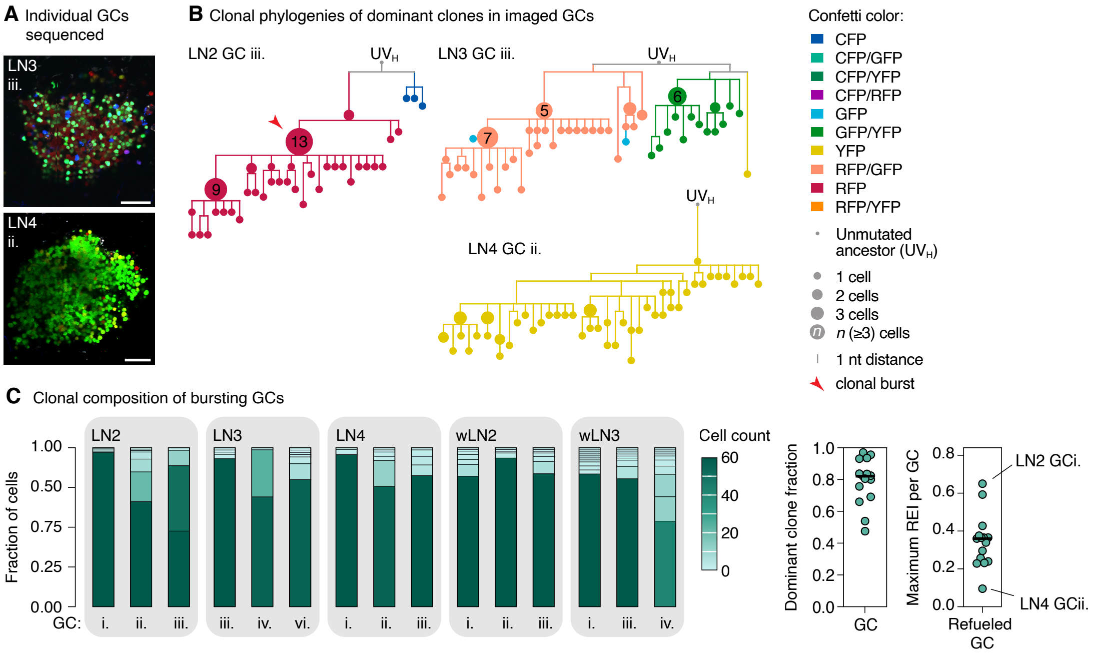


**Figure S5. Additional phylogenetic trees from sequenced GCs. (A)** Multiphoton images of GCs used to build phylogenies in (B) which were not previously represented in Figs. 2B and 4A. **(B)** Phylogenetic trees of the dominant B cell clones in the GCs depicted in panel (A), Fig. 2B, and Fig. 4A. Clonal bursts (defined as expanded nodes (> 1 cell) with REI > 0.25 [41]) are indicated by red arrowheads. **(C)** Clonal distribution of fate-mapped B cells in individual GCs. Each segment represents one clone, colored by the number of cells within the clone. The size of the dominant clone and the maximum REI in all 15 GCs are shown to the right, each symbol represents one GC.

bioRxiv preprint doi: https://doi.org/10.64898/2026.05.26.728008; this version posted May 29, 2026. The copyright holder for this preprint (which was not certified by peer review) is the author/funder, who has granted bioRxiv a license to display the preprint in perpetuity. It is made available under a CC-BY 4.0 International license.

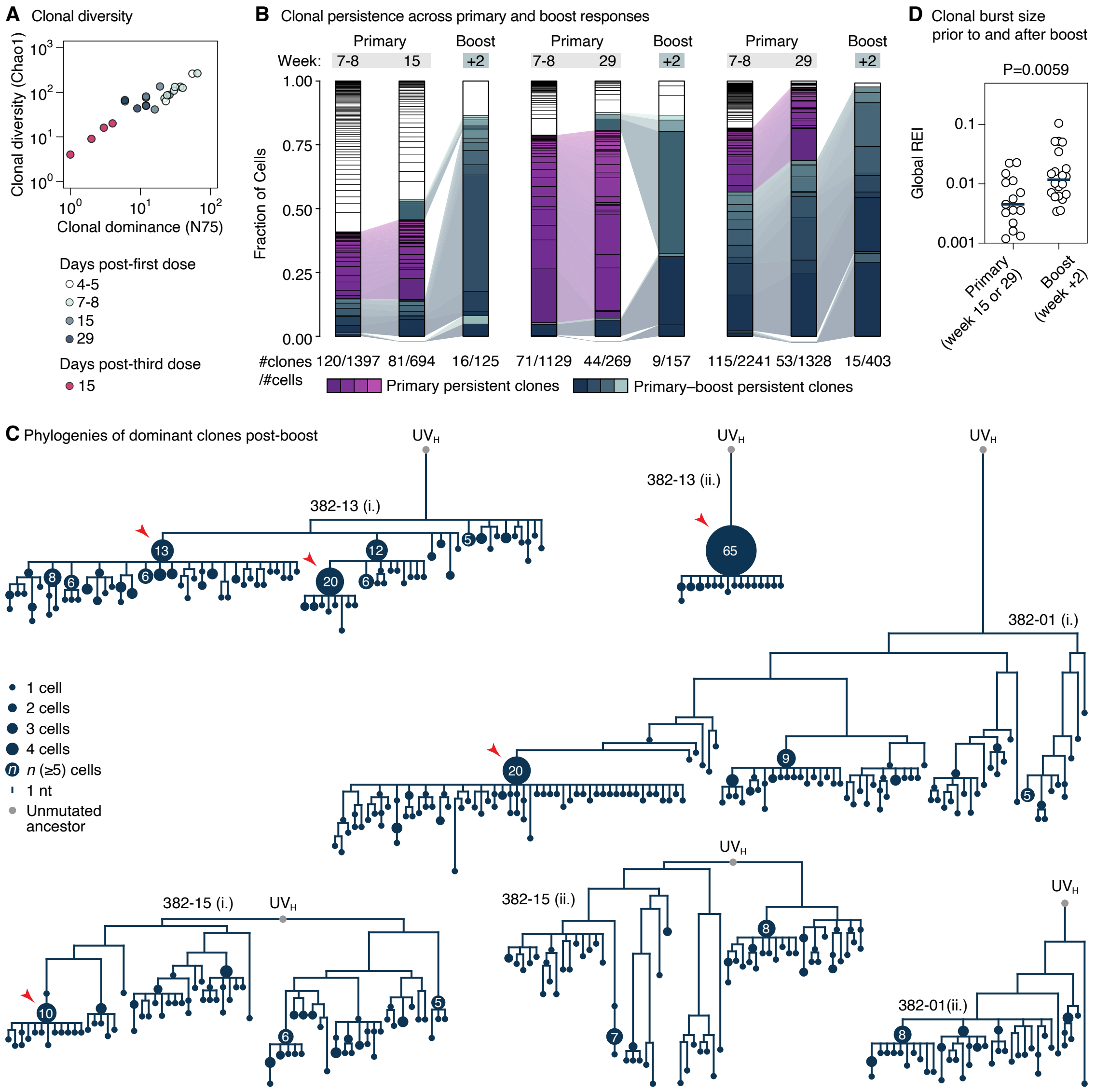


**Figure S6. Clonal persistence and expansion in human post-refueling GCs. (A)** Clonal diversity (Chao1 and N75) among spike-binding B cells. Includes data for all subjects in the dataset who had a post-boost FNA sample containing ≥ 50 GC B cells. **(B)** Clonality maps tracking the evolution of spike-binding GC B cells in human LN FNA samples at different time points prior to and after two primary-series (week 0, 3-4) and one booster (9-10 months) SARS-CoV-2 spike mRNA-LNP immunizations [25, 42]. Each segment represents one clone. Clones present in more than one compartment are connected and depicted in color; white clones were present in a single time point only. Numbers below each bar are (number of clones/number of cells) in each sample. Only subjects with two pre-boost time points and > 50 spike-binding cells in the post-boost sample were included, restricting the analysis to 3 subjects. **(C)** Clonal phylogenies for the 2 largest clones from each of the three subjects analyzed in (A), regardless of antigenic specificity. Arrowheads indicate burst points containing ≥ 10 cells in the parental node. **(D)** Global REI scores comparing clonal expansion sizes in pre- and post-refueling samples. Includes all clones containing ≥ 20 total cells and accounting for ≥ 2.0% of cells in their respective sample; samples as in (C). P-value is for Mann-Whitney U test.

bioRxiv preprint doi: https://doi.org/10.64898/2026.05.26.728008; this version posted May 29, 2026. The copyright holder for this preprint (which was not certified by peer review) is the author/funder, who has granted bioRxiv a license to display the preprint in perpetuity. It is made available under a CC-BY 4.0 International license.

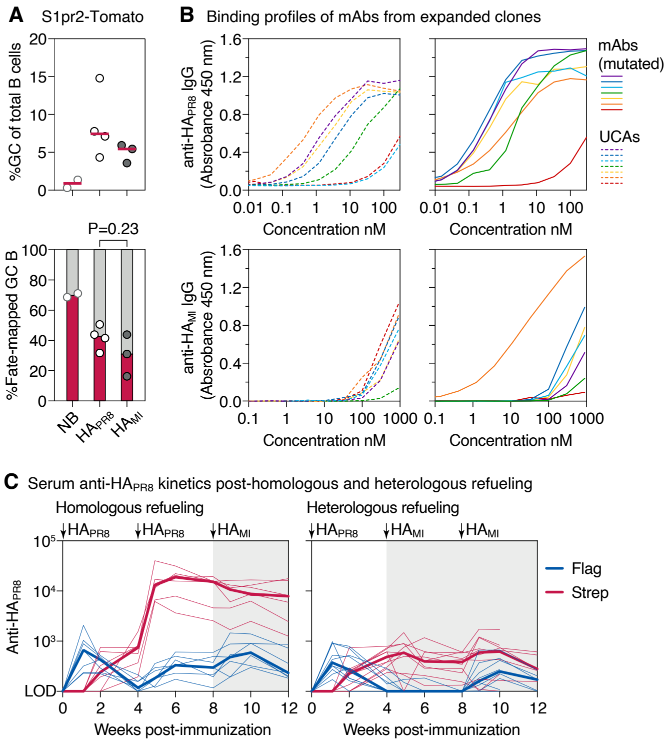


**Figure S7. Heterologous refueling with an influenza hemagglutinin variant.** **(A)** Quantification of flow cytometry (representative plots in Fig. S1B,C) of LN samples generated as in (Fig. 1A) using S1pr2-Tomato mice and upon homologous or heterologous HA refueling. GC size (top) and fraction of fate-mapped cells (bottom) are shown. Each symbol represents one mouse. P-values are for Mann-Whitney U test, only relevant comparisons are shown. **(B)** Binding to the indicated HA variant by ELISA of mAbs derived from expanded GC B cell clones sequenced from heterologously refueled mice. Summarized in Fig. 5D. **(C)** Tag-specific anti-$HA_{PR8}$ serum titers in mice fate-mapped after the primary immunization (left) or at the time of refueling (right), measured by ELISA. Thin lines represent individual mice, thick lines link medians of log transformed titer values at each time point. Anti-$HA_{MI}$ titers are shown in Fig. 5F.

bioRxiv preprint doi: https://doi.org/10.64898/2026.05.26.728008; this version posted May 29, 2026. The copyright holder for this preprint (which was not certified by peer review) is the author/funder, who has granted bioRxiv a license to display the preprint in perpetuity. It is made available under a CC-BY 4.0 International license.

## OTHER SUPPLEMENTAL MATERIAL

**Table S1. Reagents used for flow cytometry**

| Specificity | Conjugation | Clone | Source |
|---|---|---|---|
| B220 | BV785 | RA3-6B2 | BioLegend |
| CD138 | BV650 | 281-2 | BioLegend |
| CD38 | APC or BUV396 | 90 | BioLegend |
| Fas (CD95) | PE-Cy7 | Jo2 | BD |
| Streptavidin | BV421 and APC | | BioLegend |
| TCRβ | APC-Cy7 | H57-597 | BioLegend |
| Spike (Wuhan-Hu1) | Streptavidin | | Sino Biologicals |
| $HA_{PR8}$ | Streptavidin | | Home-made |
| $HA_{MI}$ | Streptavidin | | Sino Biologicals |

**Movie S1 (online only).** Z-dive through GCs from cortical to medullary. Scalebar, 50 µm; slices are 10 µm apart.

**Movie S2 (online only).** 3D rendering of sequentially imaged GCs showing pre- and post- refueling states. Scalebar, 50 µm.

**Spreadsheet S1 (online only).** Sequences of primers used for *Ig* sequencing.

**Spreadsheet S2 (online only).** Sequences of HA variants used for mRNA-LNP production.
